# MFAM: Multiple Frequency Adaptive Model-Based Indoor Localization Method

**DOI:** 10.3390/s18040963

**Published:** 2018-03-24

**Authors:** Jure Tuta, Matjaz B. Juric

**Affiliations:** Faculty of Computer and Information Science, University of Ljubljana, Vecna pot 113, SI-1000 Ljubljana, Slovenia; matjaz.juric@fri.uni-lj.si

**Keywords:** adaptive localization, indoor positioning, model-based localization, multi-frequency localization, propagation modeling, IEEE 802.11ah

## Abstract

This paper presents MFAM (Multiple Frequency Adaptive Model-based localization method), a novel model-based indoor localization method that is capable of using multiple wireless signal frequencies simultaneously. It utilizes indoor architectural model and physical properties of wireless signal propagation through objects and space. The motivation for developing multiple frequency localization method lies in the future Wi-Fi standards (e.g., 802.11ah) and the growing number of various wireless signals present in the buildings (e.g., Wi-Fi, Bluetooth, ZigBee, etc.). Current indoor localization methods mostly rely on a single wireless signal type and often require many devices to achieve the necessary accuracy. MFAM utilizes multiple wireless signal types and improves the localization accuracy over the usage of a single frequency. It continuously monitors signal propagation through space and adapts the model according to the changes indoors. Using multiple signal sources lowers the required number of access points for a specific signal type while utilizing signals, already present in the indoors. Due to the unavailability of the 802.11ah hardware, we have evaluated proposed method with similar signals; we have used 2.4 GHz Wi-Fi and 868 MHz HomeMatic home automation signals. We have performed the evaluation in a modern two-bedroom apartment and measured mean localization error 2.0 to 2.3 m and median error of 2.0 to 2.2 m. Based on our evaluation results, using two different signals improves the localization accuracy by 18% in comparison to 2.4 GHz Wi-Fi-only approach. Additional signals would improve the accuracy even further. We have shown that MFAM provides better accuracy than competing methods, while having several advantages for real-world usage.

## 1. Introduction

Internet of Things (IoT) platform predicts ubiquitous systems with every device being connected to the network to communicate with the surrounding devices. Communication and interaction between the devices in our homes, public spaces, smart cities, and offices will ease our lives by automating everyday tasks and by seamlessly sharing information between the devices. One of the most important properties of the IoT device, which is often beneficial to the other devices, is the device’s precise position in the indoor space. Most of the indoor localization methods based on wireless RF signals utilize Wi-Fi network, because it is commonly available indoors to provide wireless Internet access.

Usage of the 2.4 GHz Wi-Fi for indoor localization usually requires additional devices to be installed; for the Internet access usually one access point (AP) in the medium-sized apartment suffices, for the implementation of the indoor localization at least three APs are required due to the triangulation principle of the indoor localization methods. Other signals in the indoor space should be considered for the localization process; nowadays, higher frequency 5 GHz Wi-Fi is commonly available indoors. This frequency is rarely used in the indoor localization as higher-frequency signal is worse at penetrating walls and therefore it is more difficult to provide coverage of at least three 5 GHz APs throughout the indoor space. Newer Wi-Fi standards, which are still in the beginnings of wide market adoption, are IEEE 802.11ad and 802.11ah. The former is a new high-speed high-frequency Wi-Fi standard that promises multi-gigabit transfer speeds. Due to its frequency of 60 GHz signal typically cannot penetrate walls, therefore indoor localization method can benefit from detection of an 802.11ad signal emitted by specific AP. If such signal is detected at a mobile terminal (MT), room of the MT can be identified with high accuracy, if information about position of the walls is included in the method. Therefore, architectural aspects (e.g., placement of the walls) are important for the indoor localization methods; most the today’s methods do not include architectural information. Our proposed method includes data about placement and type of the walls in the indoor space. The 802.11ah standard is a new Wi-Fi standard designed for the IoT; low-frequency 900 MHz signal provides Wi-Fi connectivity at longer ranges, while lowering the energy requirements of the traditional 2.4 GHz Wi-Fi. The consequence of low-frequency is reduced real-world-achievable transfer speed. 802.11ah is designed for the IoT, because the majority of the IoT devices do not require high-bandwidth connections and high data throughput.

If we look at wireless communication protocols past the Wi-Fi, we can find wireless automation systems that are installed in modern homes. These systems usually rely on wireless communication protocols other than Wi-Fi, mostly due to its high-power demands. Frequency spectrum is regulated by the International Telecommunication Union (ITU) and local governments, therefore frequencies used in home automation systems differ by the regions. For example, many lighting solutions utilize ZigBee Light Link standard [1] (e.g., Philips Hue, Osram Lightify, etc.), which uses 2.4 to 2.5 GHz band. This band, by ITU specification [2], should be available worldwide without additional restrictions by the local governments. Many heating and security solutions usually utilize protocols on lower frequencies in order to avoid interference with frequency-crowded 2.4 to 2.5 GHz spectrum. Danfoss Living system utilizes z-wave protocol, which in North and South America operates on 902 to 928 MHz industrial, scientific and medical (ISM) radio bands. In Europe, it utilizes short range device band—SRD860 (863 to 870 MHz) specified by the ECC Recommendation 70-03 [3]. Honeywell’s Evohome and eQ-3’s HomeMatic are two separate automation systems sold only in Europe, which communicate via their custom protocols on SRD860 bands.

To utilize the increasing number of frequency bands typically used indoors to simplify and improve the accuracy of indoor localization, we have designed MFAM (Multiple Frequency Adaptive Model-based localization method), an indoor localization method, which uses multiple frequency bands simultaneously. It is based on the physical model of signal propagation and can detect and adapt to the changes in the environment that influence signal propagation. This way we have achieved for our method to be much less sensitive to the changes that can happen indoors, such as changing or moving the furniture. Moreover, the adaptiveness of the method addresses issues of moved wireless devices and access points, which also affect signal propagation. Therefore, our method addresses real-world requirements for indoor localization better than the other methods.

Furthermore, with our method we have successfully reduced the number of required APs. Typically, indoor localization requires many devices emitting or receiving wireless signals. For example, for wireless Internet we need one Wi-Fi AP to cover a room. For indoor localization, we must typically provide coverage of at least three APs in the same area, just due to the triangulation principle. Localization methods typically require even more APs for better accuracy. Our indoor localization method utilizes multiple signal frequencies, and therefore requires considerably less APs of a specific type for the same localization accuracy.

MFAM builds upon our self-adaptive 2.4 GHz Wi-Fi-only localization method presented in [4], which is purely model-based. The proposed MFAM method is wireless-signal- and frequency-agnostic, and it achieves 6% better accuracy when compared to the results that are presented in [4], while utilizing signals and devices that are already present in the indoors, therefore reducing needed hardware requirements and easing deployment. We have evaluated proposed method using two different frequency bands—2.4 GHz Wi-Fi and a home automation system. Due to unavailability of hardware supporting the 802.11ah standard on the market, we have chosen wireless communication system with similar frequency. The evaluation was performed in a real-world environment of a modern two-bedroom apartment.

Indoor localization has been a popular research topic in the past decade. A recent detailed state-of-the-art review paper was published by Yassin et al. [5], which contains an in-depth description of different types of approaches to indoor localization. A brief overview of the most relatable works is presented in this section.

We can divide RSSI (received signal strength indicator) based indoor localization methods into two categories—fingerprinting and model-based techniques. Fingerprinting methods, such as [6,7], require time-consuming offline procedures to survey and measure the RSSI at different site locations. During localization phase measurements from the mobile terminal are compared to the fingerprinting measurements and the location is calculated based on similarity. Offline procedure has to be done frequently as shown in [4], which makes these methods difficult and costly to implement in real world systems.

Model-based methods, such as [8,9,10] model the propagation of the Wi-Fi signals through space and obstacles, thus eliminating the need for offline sampling. Paper written by Du et al. [10] proposes a Wi-Fi method where devices are placed in the known positions that act as anchor points. Propagation model is built based on the readings from these devices. Their paper has one severe disadvantage in comparison to our method—it requires mobile terminal to emit signals to localize itself; this reduces real-world applicability in crowded spaces, as many devices trying to localize at the same time would result in signal collisions and heavy interference.

There was some research on model-based approaches utilizing frequencies other than Wi-Fi. Paper [11] describes indoor positioning system utilizing z-wave signals. In contrary to our solution, it presents a single-frequency method that also requires the mobile terminal to emit signals. Similarly, paper [12] uses emitted ZigBee signals for indoor localization. In contrast to our work it requires a difficult and time-consuming initial setup phase, in which a larger number of receivers (1 device per 1 m^2^) is used; this procedure must be redone when rearranging the indoors, thus this method is impractical for quickly changing environments.

Recent paper by Yoon et al. [13] describes radio FM-based indoor localization method. Their method does not require fingerprinting and due to the long range of the FM band results in lesser accuracy than methods based on RF signals with shorter range. When compared to our work, the method provides one order of magnitude worse accuracy while using similar number of signals and not optimizing the results with walking patterns. If such accuracy is satisfactory, it has benefits in comparison to short-range RF localization methods, because APs (FM radio towers) are public infrastructure and therefore their usage eliminates the cost of deployment and maintenance of the APs.

One of the signals often used by the authors of non-Wi-Fi indoor localization methods is Bluetooth, especially with the introduction of power-efficient Bluetooth Low Energy (BLE) specification. We can find such examples in [14,15]. Zhuang et al. in [14] combined fingerprinting and model-based approach to BLE-based indoor localization. Their proposed method has slightly better accuracy than ours, although fairer comparison could be made if their evaluation would include rooms and not only hallways of the building. Methods that combine Wi-Fi and Bluetooth signals have been researched, such as [15], although we must note that Bluetooth and 2.4 GHz Wi-Fi work on similar carrier frequencies. Hossain el al. [15] showed that their Bluetooth-only localization has approximately the same accuracy than the combination of Bluetooth and Wi-Fi.

Laufer et al. [16] reverse engineered parts of the HomeMatic wireless protocol while trying to execute an attack on it. Their work is significant for our research, because they showed detailed information about the underlying protocol, which we use for the evaluation of our method.

The paper is structured as follows: Section 2 presents the proposed method. Following two sections provide details on the evaluation and present results. Section 5 provides the discussion and interpretation of the localization results. Section 6 concludes this paper and gives thoughts on further research directions.

## 2. Proposed Localization Method

In our past work [4], we have defined a 2.4 GHz Wi-Fi method that utilizes a model based on theoretical propagation of a wireless signal. The parameters of the propagation model have been inferred from the beacon packets of the Wi-Fi detected at the APs. Localization method can calculate the missing parameters of the propagation model by knowing the architectural properties of the building plan, positions of the APs, and signals that are captured between APs. This process has enabled our Wi-Fi propagation model to constantly adapt to the most recent changes in the building that influence the propagation of the signals, thus achieving better accuracy and making the model more suitable for real-world usage.

The primary motive for developing multiple frequency localization method lies in the future Wi-Fi standard—the 802.11ah, also known as “Wi-Fi HaLow”. We are convinced that this standard in combination with ever-present 2.4 GHz Wi-Fi can achieve localization accuracy and real-world practicality needed for the IoT; not to mention the benefits that could be achieved by including 5 GHz or 60 GHz Wi-Fi.

Nowadays, APs and wireless adapters supporting HaLow are not yet commercially available; to develop a method for the future wireless standard we began looking at other wireless communication standards. We have decided to develop our multiple frequency method by utilizing 2.4 GHz Wi-Fi and a home automation system that utilizes the SRD860 band. This band is appropriate as frequencies are similar to the Wi-Fi HaLow’s 900 MHz band, and therefore we can expect similar effects on the propagation in the real-world setting; selected home automation system HomeMatic communicates over the frequency of 868 MHz. Usage of a non-Wi-Fi-based network poses challenges as these networks are topologically and communicationally different. In the following subsections we will present the MFAM method and the challenges we faced while implementing the method to the home automation network.

### 2.1. MFAM: Multiple Frequency Adaptive Model-Based Localization Method

**Figure 1:**
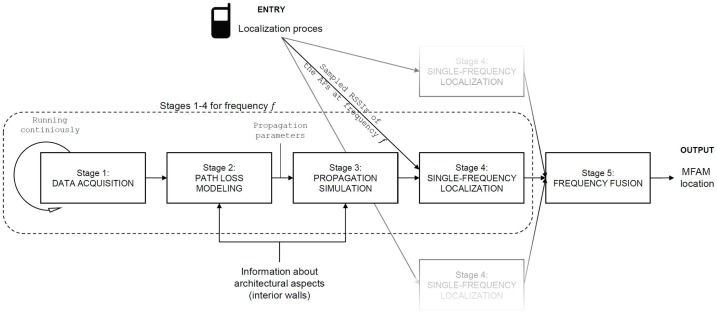
Overview of the method and its five stages.

The MFAM method is divided into five stages: data acquisition stage, path loss modeling stage, propagation simulation stage, single-frequency localization stage, and frequency fusion stage, which can be seen presented in the Figure 1. The following subchapter will guide the reader through all five stages of the method.

In the data acquisition stage, we monitor the signal propagation by observing RSSI information of packets that were captured at the available APs. These packets have traveled the space between two individual APs and therefore are influenced by scattering, room properties, obstacles, etc. By monitoring these packets, we get live information of the signal propagation through real-world indoor space. We can capture each RSSI as ${\mathrm{RSSI}}_{j,i}$ where j is labeling $AP_{j}$ at which signal was measured and i stands for $AP_{i}$ from which the measured signal was emitted. Data acquisition stage is constantly running in the background and gathering live data, from which we infer current parameters of signal propagation.

In path loss modeling stage, we derive propagation parameters from measurements acquired in the data acquisition stage and selected propagation model. We based our path loss model on the logarithmic-distance path loss model for the free space propagation of RF signals [10]:

(1) $$PL=PL_{0}+10\gamma \;\log_{10}\frac{d}{d_{0}}+\Delta$$

We further extended the model with the effects of the walls on the signal propagation. Firstly, we included a value that is influenced by the number and the type of the penetrated walls. Secondly, we included a parameter that accounts for the relative position of the transmitting AP and the closest wall, which acts as an antenna reflector. We have especially observed this influence in the line-of-sight scenarios; in cases where there are more dividing walls, the effect of this parameter slowly diminishes. If we write a system of equations for such extended model between three points, one between i and m and second between i and n, and then produce difference between them we get Equation (2). Collecting the last 15 min of data from the data acquisition stage produces an over-determined system of equations based on (2).

(2) $$\left(RSSI_{m,i}-W_{m,i}\right)-\left(RSSI_{n,i}-W_{n,i}\right)=10\gamma_{i}\;\log_{10}\frac{d_{i,m}}{d_{i,n}}+10\beta_{i}\;\log_{10}\frac{d_{i,m}}{d_{i,n}}\times \left(\alpha_{i,m}-\alpha_{i,n}\right)$$

In (2), $W_{j,i}$ stands for the impact of the walls between $AP_{j}$ and $AP_{i}$, which we can calculate from the architectural map of the indoors. $\gamma_{i}$ is path loss exponent for $AP_{i}$, f is signal’s frequency, and $d_{i,j}$ is the distance between APs with respective indexes. $\alpha_{i,j}$ is angle in radians between normal vector to the closest wall of $AP_{i}$ and direction of direct distance between $AP_{i}$ and $AP_{j}$, divided by π/2. Parameter $\beta_{i}$ is describing the effects of angle $\alpha_{i,j}$ on propagation. In the end of path loss modeling stage, we get parameters that define the propagation model for the AP (i.e., $\gamma_{i}$ and $\beta_{i}$). As can be seen, only the measured RSSI values in the last 15 min before localization takes place influence the result. Consequently, our method does not rely on static assumptions of the propagation parameters, but infers the current parameters of the signal propagation model by monitoring the packets that have traveled the space between the APs. Usage of the difference of the RSS signals ensures that RSSI readings are comparable between different devices. This had to be done, as absolute values are not necessary comparable per 802.11 standard [17,18].

In propagation simulation stage, we use the previously inferred parameters of the propagation model to simulate the signal propagation and build a precise propagation map on the localization server. We utilize ITU propagation model, which we have extended with β parameter and can be seen in (3); $PL_{i,j}$ stands for path loss between $AP_{i}$ and selected point j. Propagation map is built on the server and is updated when new set of propagation parameters is defined in path loss modeling stage.

(3) $$PL_{i,j}=20\;\log_{10}\left(f\right)+\gamma_{i}\;\log_{10}\left(d_{i,j}\right)+\beta_{i}\times \alpha_{i,j}\times \log_{10}\left(d_{i,j}\right)+W_{i,j}-28$$

During the single-frequency localization stage, mobile terminal (e.g., mobile phone, IoT device) captures multiple RSSIs from the signals emitted by neighboring APs. RSSIs are firstly filtered in order to remove the outliers in highly variable RSSI data. After filtering, we normalize RSSI values to the relative values of the strongest captured signal to avoid the incomparability of absolute values of RSSI readings. Similarly, the propagation map from the propagation simulation stage is altered to express the values relative to the strongest RSSI. We get the position of the mobile terminal by finding the point of the map, which has the lowest least-square error when comparing the propagation map and the readings from the mobile terminal. Output from the single-frequency localization stage is the position estimation based on single frequency.

In the frequency fusion stage, we are fusing location outputted by all single-frequency localization stages by weighted average. Weights are defined as reciprocal values of the standard deviation. Final MFAM location is therefore calculated by (4), where ${\left(x,y\right)}_{\mathrm{MFAM}}$ is a location that is based on multiple frequencies, f is specific frequency, ${\left(x,y\right)}_{f}$ is the location based on the chosen frequency, and $SD_{f}$ is the standard deviation of the method at the chosen frequency.

(4) $${\left(x,y\right)}_{\mathrm{MFAM}}=\frac{1}{\sum_{f}\frac{1}{SD_{f}}}\left(\underset{f}{\sum }\frac{1}{SD_{f}}{\left(x,y\right)}_{f}\right)$$

When combining multiple frequencies for localization, we must consider the accuracy at each individual frequency. Averaging the results for multiple frequencies could result in a worse performance than using a single frequency. We can deduce the combining method in terms of statistical measures of accuracy and precision. The precise method will always output approximately the same value for the same input parameters, which is not necessarily close to the real-location (in this case it is inaccurate). The accurate method will output the location close to the real-location, although locations can be scattered (in this case we call it imprecise). In our case, due to different signal frequencies used, difference in propagation at these frequencies, etc., precision of single-frequency localization varies. In the distribution of values, precision is measured by standard deviation. The higher the precision, the smaller the standard deviation, and vice-versa. Therefore, we have weighted the outputs from the single-frequency localizations by the reciprocal value of standard deviation. Because it is impossible to estimate in advance the value of standard deviation for a specific frequency at a specific location, it should be determined empirically.

### 2.2. Gateway-Based Networks

We have designed MFAM with the specific objective to use multiple frequency signals to improve accuracy and simplify the requirements regarding the equipment. We are interested in OSI Layers 1 and 2 of the communication protocols. These layers ensure the physical transmission of the signals and provide means of control; we leverage them to get the RSS/RSSI. Nowadays, when algorithms are run on servers (cloud-based infrastructure) it is trivial to communicate with devices that are connected to the IPv4/IPv6 network. Wi-Fi devices are usually always connected to the IP network and therefore we can connect to each device from the server on which localization algorithms are running. On the other hand, using networks with devices not directly connected to the IP network for indoor localization is much more challenging. Such networks can include home automation systems, Bluetooth networks, ZigBee, etc., and often facilitate multiple devices, which communicate over some proprietary protocol. Only one device is usually connected to the IP network and acts as a gateway or base station. This device is the only addressable device from the localization server over the IP protocol; direct communication with other devices is not possible. We will call such networks gateway-based networks. There are specifics in each gateway-based network but the steps to RSS-based indoor localizations are similar. In this subsection, we focus on home automation systems, because we utilize those in our evaluation environment, however the findings are valid for all types of gateway-based networks.

To infer the parameters of the propagation model of a Wi-Fi network, we can connect to each AP and gather information about the RSSI of the signals, which are emitted from the devices in range. While using gateway-based networks, we have no means to connect to specific device. Consequently, we cannot get information about received packets. It is a usual practice in the home automation system for the battery-powered devices to periodically send status packets to the base station. Such packets are important for the base station as it must know which devices are in its range, their state (e.g., open/closed switch, high/low digital sensor value, etc.), and properties, such as battery status. Base station usually receives these packets and stores their information, while also recording the RSSI. These recordings provide us the information required to calculate the parameters for our propagation model.

The rate of possible RSSI measurements also differs in the two different network types. In the Wi-Fi network, the only thing bounding the rate of this information is the time that is needed for a single scan and setting the beacon-packet interval for the network. This results in a possibility of having RSSI information recorded at specific AP multiple times per minute. Having so frequent data to infer the state of the wireless network allows for us to calculate the propagation parameters for each AP separately. Inferred parameter set for a single AP therefore exaggerates the effects of AP placement, room shape and obstacles in AP vicinity, which all have influence on the signal propagation [19]. Battery-powered home automation devices usually save on energy by emitting status packets every few minutes. Due to much less data from which propagation parameters can be inferred, it is not sensible to calculate the parameters of propagation for each device separately, but rather to calculate a single set of parameters for the propagation model at this frequency. Therefore, parameters exaggerate much less specific properties of a single AP, but represent the averaged propagation model for signals of a specific frequency.

When a mobile terminal tries to define its position, it must measure the RSSIs to the APs that are in reach. In the Wi-Fi network, mobile terminal scans for the beacon packets, which are periodically emitted. In the gateway-based networks, there are usually no beacon packets, which would announce the presence of a network. Therefore, we have utilized the already described status packets for the mobile terminal to scan and measure the RSS.

Due to the lack of the research on propagation of the signals through the walls at frequencies that are used by different gateway-based networks, we could not define the impact of the walls on the signal propagation based on past research. Rather we had to define them experimentally ourselves, as described in the “Evaluation protocol” and “Results” sections of this paper. Resulting values are the initial values for our system. Due to the adaptive nature of our method, method adapts automatically and overcomes the potential errors induced by these parameters.

For the majority of home automation systems, a mobile terminal has to be kept in an evaluation point for a longer period of time. This is induced by the gateway-based networks, the rate of status packets sent by them, and the number of status packets that are needed by our method. Manufacturers usually define the communication protocol and the rate of the status packets with the aim of preserving battery power in devices, as discussed in [16]. In devices that are constantly connected to the mains power (e.g., lighting solutions, relay devices, etc.), this deficiency could be easily overcome by the manufacturers, which would further improve the accuracy of our localization method.

## 3. Evaluation

This section contains detailed information about the evaluation of our method. The first subsection contains a detailed description of the evaluation environment, which is important for making the evaluation repeatable. The following subsections describe the evaluation equipment and the evaluation protocol, while the next section contains the evaluation results.

### 3.1. Evaluation Environment

**Figure 2:**
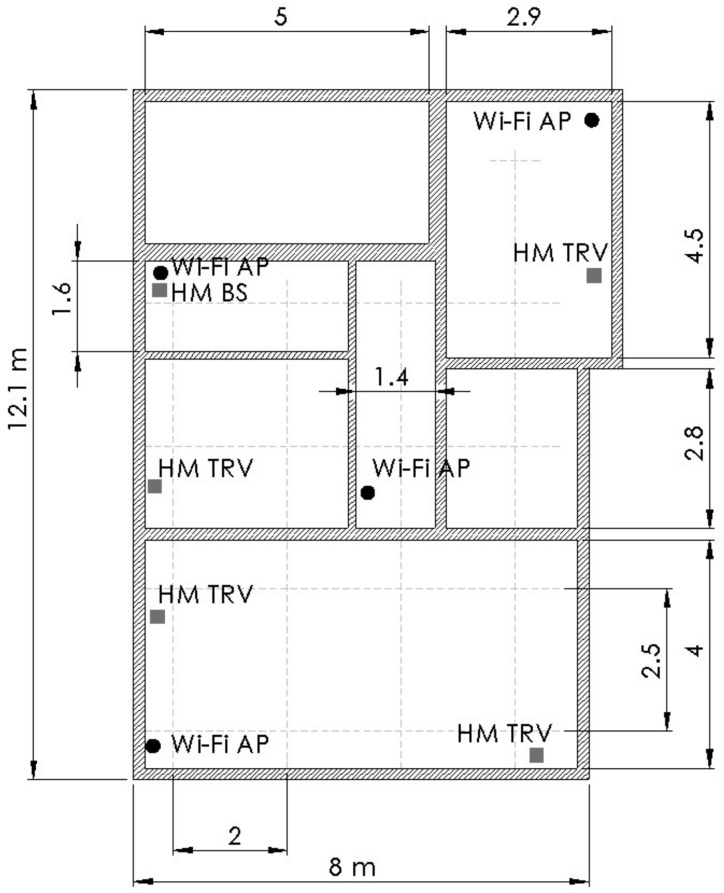
Evaluation environment with marked positions of Wi-Fi access points (Wi-Fi AP), HomeMatic thermostatic valves (HM TRV) and HomeMatic base station/gateway (HM BS). Evaluations points are marked by crossings of dashed gray lines; unit of length is meter [m].

We have selected a real-world evaluation environment—a modern two-bedroom apartment constructed in 2009. It has six rooms and a mixture of brick and plaster walls. Room sizes range from 5 to 30 m^2^. Apartment has been equipped with real-world furniture and fixtures used by a young family. We have set up a 2.4 GHz Wi-Fi network consisting of four APs. We know from our previous work [4] that the placement of the APs in the corners of the area of the interest is usually the optimal placement. For this evaluation, we have chosen sub-optimal conditions to better reflect real-world scenarios and set one Wi-Fi AP in the middle of the apartment and three in the corners of the area of interest, as shown in Figure 2. All of the APs were connected to the router via wired Ethernet network. Because the evaluation is in a real-world indoor space, the Wi-Fi network was not isolated. At any given moment, we could detect between 10 and 20 different Wi-Fi networks in range. The positions of the APs differ due to the constraints opposed by the real-world furniture setting. They ranged from positions on the table 0.5 m above ground to the above-closet locations at 2.3 m.

We have chosen to use HomeMatic as the home automation system, as discussed before, its frequency of 868 MHz is similar to the 900 MHz that is used by Wi-Fi HaLow. HomeMatic is popular in Europe as it includes devices for thermal control (heating and cooling), lighting (dimmers, relays, switches), security (alarms, sensors, door locks), and other devices, such as smoke detectors, etc. The home automation system in our evaluation consists of a base station (in Figure 2 marked by “HM BS”) connected to wired Ethernet network, a wireless relay, and four digital thermostatic valves. We could not influence the position of these devices in our evaluation environment. Our system is not the only one using SRD860 frequency spectrum in the evaluation environment as we could detect HomeMatic packets originating from neighboring apartments. To ensure the approximately constant transmitting power of HomeMatic devices, we have used only readings from thermostatic valves, which are in Figure 2, marked as “HM TRV”. We have eliminated signals from the wireless relay because it is powered from the mains power (opposed to battery powered digital thermostats), and therefore it has different operational mode, especially regarding the frequency of sent status messages and transmitting power [16]. HomeMatic systems emit status packets approximately every 3 min. Thermostatic valves were all positioned between 0.3 and 0.6 m above ground; two of them were positioned without direct obstacles, two of them were behind furniture, and were therefore heavily obstructed.

### 3.2. Evaluation Equipment

For the IoT device preforming indoor localization, we have chosen a Raspberry Pi 2 based terminal. In each of the evaluation points, a mobile terminal was positioned. The configuration of the mobile terminal has been a Raspberry Pi 2, battery, Wi-Fi USB dongle (commercial name WIPI), and a HomeMatic communication module (commercial name HM-MOD-RPI-PCB), which communicates with Raspberry Pi 2 via UART interface.

During the localization process, the mobile terminal was held approximately at head-height position. We have captured RSSI values at Wi-Fi APs, with a “iwlist [interface] scan” command build into the Linux distribution. RSSIs of HomeMatic devices were captured using Homegear software (version 0.6.7), which we installed and configured on the Raspberry Pi. Homegear software utilizes HM-MOD-RPI-PCB to capture HomeMatic packets and their respectful RSSIs.

### 3.3. Evaluation Protocol

During the evaluation design, we determined 17 evaluation points in the apartment. Points were arranged in a mesh as depicted in Figure 2—crossings of dashed gray lines represent the evaluation points. Spacing between the evaluation points is 2 m and 2.5 m for the width and length of the apartment, respectively. The sampling sequence in all four evaluations was the same; we started at the lower left position (0.5, 0.5) and measured the positions in left-to-right and right-to-left pattern finishing in the top evaluation point. For the propagation simulation stage of our method, we have chosen a mesh with a uniform size of 0.5 m, which is the biggest uniformly sized mesh that fits our evaluation points.

We have performed four independent measurements in every evaluation point at different days and times, and obtained four datasets (DS). For the Wi-Fi devices, we made sure that between evaluations the devices were powered off for at least 1 h (to cool the chipset to room temperatures) and that they were powered and operational for at least 1 h before the evaluation in order to eliminate the possible thermal variations due to the initial heating that can influence the results [20].

Captured data was used to perform the analysis of positioning error. Firstly, we analyzed the accuracy in Wi-Fi-only case to show versatility and universality of our method. Secondly, we used home automation signals only to perform the evaluation and analyze the accuracy. Thirdly, we have combined both the Wi-Fi and home automation signals to obtain indoor localization based on multi-frequency signals.

## 4. Results

This section presents the results from our evaluation. To prove that our method achieves better results when utilizing two or more frequency bands, we will first present localization results using a single signal type (i.e., Wi-Fi and home automation separately). In the last subsection, we will show the results for the combined signals and present the accuracy improvement.

### 4.1. Wi-Fi Localization

For the Wi-Fi signal evaluation, the parameters of propagation that express the power loss through the walls was set as in [4], by the results obtained in [19].

**Figure 3:**
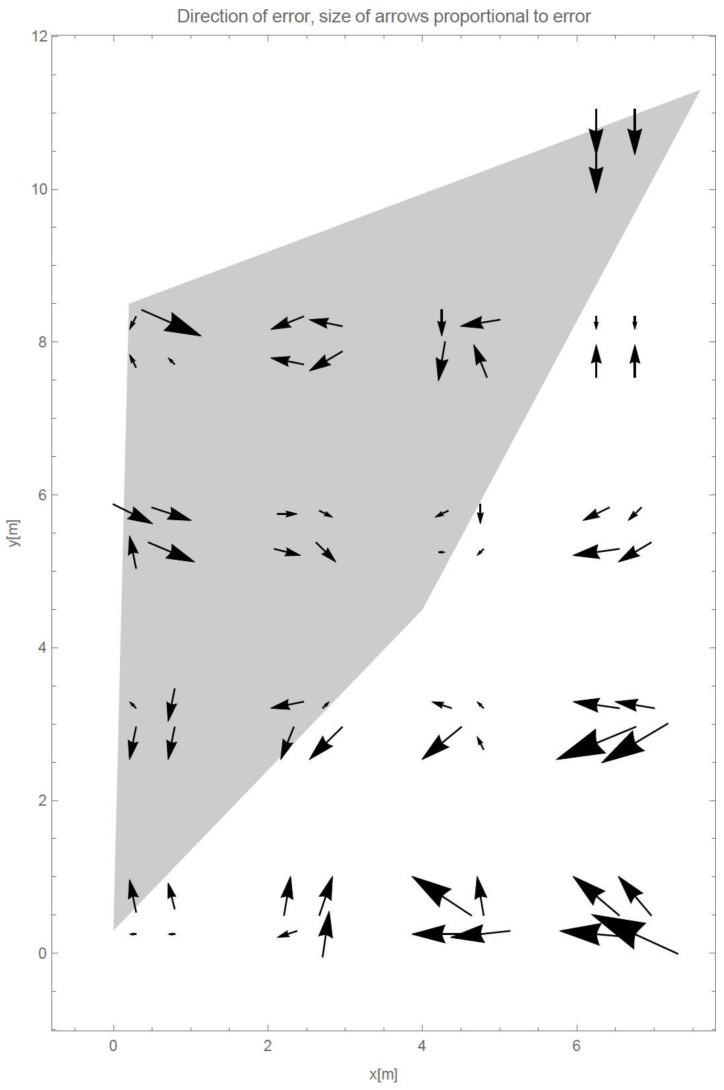
Directions and proportional size of errors for the localization of all four datasets. Gray polygon’s vertices show positions of the Wi-Fi APs.

Table 1 shows mean error, median error, and standard deviation of all four datasets. Figure 3 shows directions and proportional sizes of error in every dataset for each of the evaluation points. In the Figure 3, gray polygons depict positions of Wi-Fi access points. As can be seen from Table 1, the averaged mean error through all four datasets is 2.65 m with standard deviation of 1.51 m.

### 4.2. HomeMatic Home Automation System Based Localization

In the scenario of HomeMatic-only signals, we had to define the values of the parameters, which describe the influence of the walls on the signal propagation. Because these frequencies are not yet researched for indoor localization, no valid data can be found regarding the impact on propagation. Consequently, we had to numerically model the impact of the walls on the SRD860 signals for both of the wall types on each of the datasets and cross validate the results on other datasets. The reader should be minded that in the future when 900 MHz band will be researched thoroughly, the MFAM method should utilize values that are determined by rigors experiments, similarly as it does with the Wi-Fi signals. We do not possess the equipment or the expertise to determine the independent effects of the walls on signal propagation at selected frequency. Values obtained by the described estimation are influenced by our method, the indoor setting, amount of furniture, etc.

**Figure 4:**
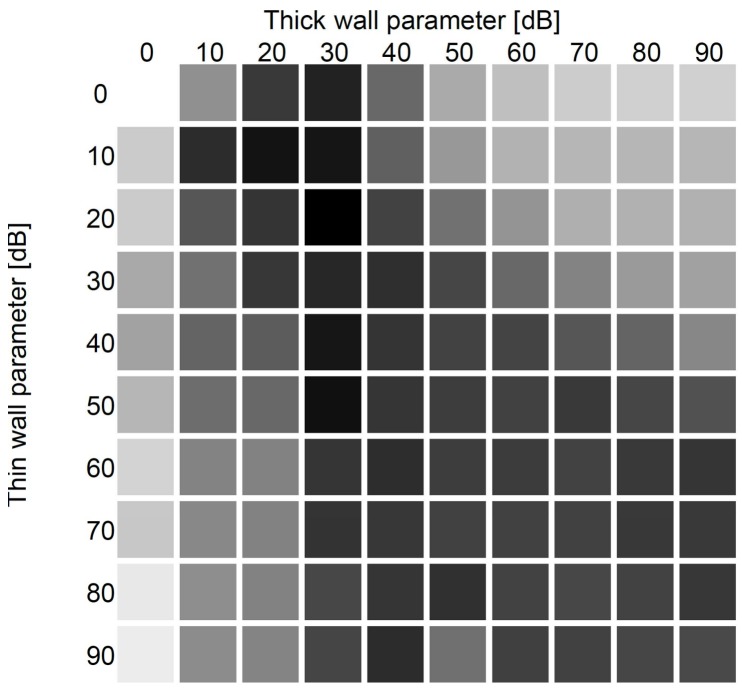
Accuracy of the method while using different parameters for the parameter describing the effect of thin, plaster walls (vertical axes) and thick, brick-and-concrete walls (horizontal axes) on the signal propagation. The darker the color, the higher the accuracy with those parameters.

The results of numerical modelling of the effects of the walls are shown in Figure 4. The darker the color at a specific point in the graph, the higher average accuracy while using those parameters. In a finer analysis, we have narrowed the parameter values. In case of thick walls in range 15 to 30 dB, and in the case of thin walls to the values in range between 0 to 21 dB; in both cases with 3 dB steps. With another set of numerical modeling of the wall effects and cross checking with the other datasets we have set the wall parameters to be 9 dB in case of thin walls and 18 dB in case of thick walls.

Table 2 shows mean error, median error, and standard deviation of all four datasets. We can see larger average and mean errors than in Wi-Fi only case, but is still in the acceptable range as described in the “Discussion” section. The average mean error is 3.21 m, which is 20% worse than in Wi-Fi-only evaluation. The averaged median error is 16% larger, while the standard deviation is 24% worse.

### 4.3. Multi-Frequency Localization

For standard deviations of the localization method using single-frequencies, as defined in “Evaluation” section, we have used averaged standard deviations from Table 1 and Table 2, because these average values are based on better and worse preforming datasets DS1 to DS4.

Table 3 presents mean error, median error, and standard deviation when we combined both of the frequency bands. We can observe better mean and median errors than in any of the previous cases. We can observe 18% and 33% better accuracy in comparison to Wi-Fi and HomeMatic average errors, respectively. We can also observe similar improvement of the standard deviation.

**Figure 5:**
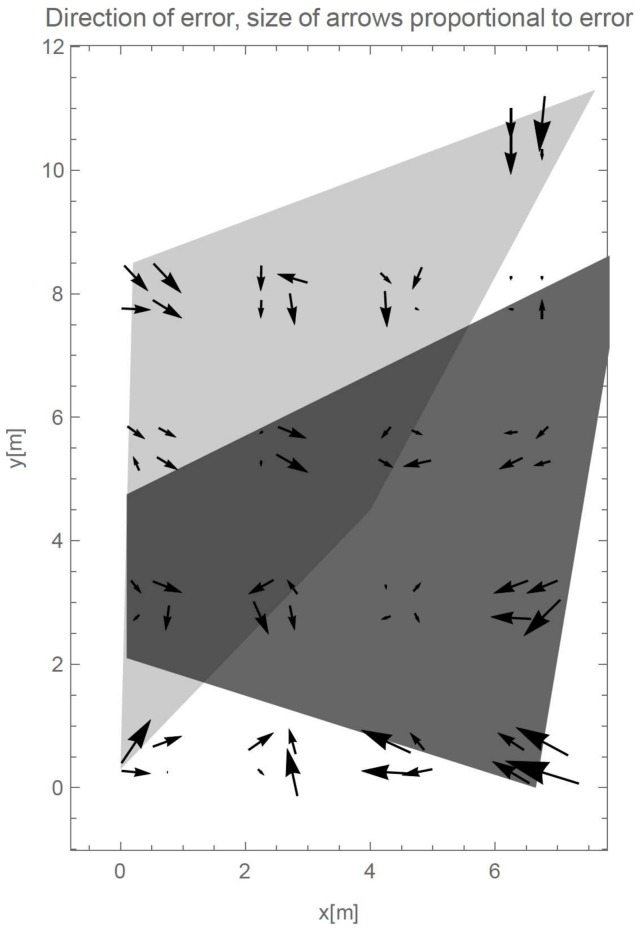
Directions and proportional size of errors for the localization of all four datasets. Light polygon’s vertices show positions of the Wi-Fi APs, dark polygon marks HomeMatic devices.

Figure 5 shows proportional error sizes and directions of the errors in all four datasets. Lite polygon (the one that is the same as in Figure 3) has vertices in the positions of Wi-Fi APs, while dark-gray polygon has vertices in the positions of HomeMatic radiator thermostatic valves (as marked in Figure 2).

## 5. Discussion

The results of the Wi-Fi only method, presented in Table 1, on all four datasets can compete with the current state-of-the-art methods with average median error of 2.59 m and standard deviation of 1.51 m. Our results are similar or better than 2–3 m accuracy, as reported by Du et al. [10], 3.5 m accuracy of Lim et al. [8], and WiFi evaluation of Tarrio et al. [9], which reported from 7 m accuracy in case of Hyperbolic based algorithm, down to 3 m average accuracy reported in case of Weighted circular algorithm. In contrast to our four AP requirement in the Wi-Fi case, evaluation of Du et al. required four APs and three additional anchors, but the evaluation has been done in a less challenging environment consisting of two long hallways. Tarrio et al. [9] evaluated the method in approximately similarly sized floorplan while using the same number of the APs and got worse best-case average localization error. Although method presented by Lim et al. has the biggest average error it has been done in approximately six times bigger evaluation environment, while requiring two times more APs.

In Figure 3, we have marked Wi-Fi APs with gray polygons. We can clearly see that the average error in localization accuracy of the points inside or close to the polygon is smaller than the accuracy of localization of the validation points far from the polygon (e.g., points in lower right of the figure). This gives us important conclusion for all similar methods—AP positions can have great impact on localization accuracy of the methods; this is also emphasized in [12]. Similarity of the results in single point in Figure 3, with some outliers, shows us that the sampling time and the sequence do not have significant impact on the method and that therefore the method is stable.

Figure 4 shows the results of a coarse optimization of wall effects in HomeMatic evaluation. In the bottom-left part of Figure 4 we can see that we get less accurate results (i.e., lighter color in Figure 4) when plaster walls are defined as having bigger impact than brick-and-concrete walls. Similarly, bad influence on the localization has neglection of the thin walls, as can be seen in the top-right part of the figure. As can be seen, even if there are only two plaster walls in the apartment, we should not neglect them. Resulting values in the lower right show good overall results, while exaggerating the impact of both wall types. In such cases, method is confined to the room, but such values result in lower accuracy in the bigger rooms. Interestingly, our modeling of the walls shows that neglecting the wall-effects (white square at top-left in the figure) gives the worst results. This further supports the fact that the inclusion of the architectural information into localization method can result in better accuracy.

Table 2 shows the evaluation accuracy of the method when only the HomeMatic devices were used. The bigger maximal errors than in the Wi-Fi signal case are expected, as we have much less data to infer the propagation parameters. In case of Wi-Fi signals, we infer path loss exponent for each access point separately, and therefore capture the influence of the AP position, scattering due to the room size and the shape much more precisely. In our setting, we calculated four different path loss exponents, each from 3×15 measurements. In the case of HomeMatic, we had to use only one (average) path loss exponent from 3×15 measurements, as discussed previously. Comparison of the Table 1 and Table 2 shows that during an evaluation DS1, we got similar accuracy in HomeMatic and Wi-Fi case; in DS2 to DS4 the performance was worse.

Finally, we have combined both of the frequency bands. The resulting mean and median errors (shown in Table 3) are outperforming both single-band methods. Since the Wi-Fi-only evaluation is comparable to the current state-of-the-art methods, our multiple frequency evaluation of MFAM shows it can rival the best existing methods. In each DS multiple frequency evaluation results in higher accuracy than both single-band methods; accuracy improvement ranges from minimum 6% (in case of Wi-Fi, DS2) to 40% (in case of HomeMatic, DS3). With multi-frequency approach, our method has achieved 17% higher average median accuracy when compared to Wi-Fi only approach, while having 14% lower standard deviation. It improved HomeMatic only-approach by 32%, while having 30% lower average standard deviation.

RSSI values, as the name suggests, indicate received signal strength (RSS) values. RSS value of wireless communication signals of any type in real world indoor setting exaggerates heavy variability due to wall reflections, multi-path effect, etc. Therefore, having four stations, which in turn means four fixed origins in the triangulation problem results in localization variability. The number of Wi-Fi points in relation to the size of the indoor area has a key influence on the accuracy. The only thing stopping us from changing odds of this ratio is real world implementation and practicality. Development of a Wi-Fi localization method with one AP in every room and evaluation in a floorplan with many small rooms would result in small mean and median error if we would only set the location of a mobile terminal to the center of a room from which we sense the AP with the highest RSSI. Utilizing 5 or 60 GHz Wi-Fi, which are worse at penetrating the walls than the 2.4 GHz, would further improve our results. Real-world usability of the localization method is the most important limiting factor to the number of Wi-Fi Aps. Therefore, we have limited ourselves to four APs for this evaluation, which is only one more than the minimum number that is required for any triangulation-based localization method. Having that one extra AP enables us to partially address the variability of the RSSI.

Figure 5 shows the direction of the errors for the multi-frequency method evaluation. Sizes of the arrows are proportional to the errors. Arrows are near the evaluation point and point towards the method’s location estimation. Near every evaluation point, there are four arrows, which correspond to the four datasets. Comparison of Figure 3 and Figure 5, while acknowledging Table 1 and Table 3, shows us that although the absolute values of the errors (as shown by the means and medians in the tables) have become smaller, the distribution of the sizes and the directions of the errors in space have stayed similar. Figure 5 shows that we can expect smaller localization errors with our method in the center of the map. This confirms that the position of the devices transmitting or receiving signal in RSSI-based localization have influence on the accuracy. Worse estimation in vicinity of the devices has its roots in the logarithmic nature of RSS propagation, therefore it is harder to estimate and/or sample RSS near the transceiver than further away.

We have shown how usage of multiple signal sources (in our case Wi-Fi and home automation system) improved the indoor location accuracy when compared to single signal methods. RSSI can be considered to be a normally distributed value [21]. Statistics of normally distributed process teaches us that having more samples to infer the normally distributed value lowers the variance of the result. Therefore, in our context, having more APs from which we could infer the propagation would theoretically give us better results (i.e., smaller variance). Disregarding real-world limitation, one could increase number of APs to for example 10 APs per room. This would result in more packet collisions and interference between signals and therefore worse wireless Internet performance; collisions would therefore limit the Internet access, which is usually the purpose of the Wi-Fi. We have therefore implemented our method to use multiple signal frequencies instead of increasing the number of APs and shown that this way we have achieved considerably better accuracy. Adding 3rd, 4th, etc. signal type would further increase the accuracy, assuming that the parameters of the RSSI distribution (and consequently single-frequency accuracy) are not significantly worse.

As already mentioned in related work section, other model-based methods exist. In contrast to our method, most them require mobile terminal to emit the signals, which are picked by the APs. In our method, the mobile terminal does not need to emit packets and to take valuable bandwidth, as it determines its location by measuring RSSI values of the incoming packets. This is particularly important in areas where many devices are trying to obtain the indoor location—e.g., factories, public spaces, commercial buildings, etc. To reduce the number of APs needed for the implementation of the localization in a specific location, our method uses multiple signals, which are already present in the buildings, which is one of the key differentiators of our work when compared to others.

If we compare results of our 868 MHz evaluation to other non-WiFi method, we can see that our method has much higher localization accuracy than FM-based method [13], which has an error in the rages of 15 m, 10 m and 6 m for the cases of no calibration, runtime calibration, and runtime calibration aided by the path matching algorithms, respectively. Our 868 MHz-only method and ZigBee-based method by Shimosaka et al. [12] were evaluated in similar floorplan, yet authors of [12] achieved 30% better accuracy, while utilizing 25 to 100% more APs, although, as discussed previously, their approach requires difficult setup phase. The reported accuracy of method presented by Hossain et al. [15] is approximately 50% worse than MFAM. The evaluation was done in a lecture room, where mobile terminal and APs are in line-of-sight. We could not find any model-based method, which would be using multiple signal with different signal bands, thus our method is one of the first model-based indoor localization methods utilizing multiple frequencies for localization. One of the recent fingerprinting approaches [22] in which Wi-Fi and Bluetooth signals were fused, achieved 15% worse accuracy than MFAM in 60% bigger evaluation space, utilizing 25% more devices.

Ruan et al. [23] developed a localization and tracking system using UHF passive RFID tags. In a residential evaluation in three rooms (approximately 45 m^2^ of combined size), 34 tags were deployed and four antennas. Although average errors of 1 m are shown, the usage of nearly one tag per square meter is in our opinion far from real-world acceptable deployment; they also show that reducing the number of tags results in bigger errors, e.g., more than 2.5 m when using approximately one tag per 2 m^2^. System Plils was developed by Li et al. [24], it utilizes 2.4 GHz signals and was evaluated in an environment of area of 24 m^2^, while utilizing 10 reference nodes and one wireless reader to track position of a robot with accuracy of 0.75 m. They emphasize on using cheap off-the-shelf wireless chip, but nevertheless such density of devices is not possible in real-world deployments. Although the results in [23,24] present better average errors of localizations, these system are not usable in the IoT enabled homes. As shown, passive RFID-only approaches do not match the accuracy of MFAM if real-world-deployment constriction of number of the tags is considered. Authors in [25] utilize seven active RFID tags in the area of 1600 m^2^, they show that RFID-only and particle filter approach cannot achieve needed accuracy, as it averages in errors of 5 m or more; after the inclusion of the dead reckoning and the inclusion of the indoor map they improved the method to achieve approximately the same accuracy as MFAM. This shows that addition of dead reckoning algorithms could improve the MFAM, but at the same time, such fusion limits the applicability of the method.

Noh et al. presented CLIPS positioning scheme [26], in which multiple individuals are moving in the indoors, for which the floorplan is known. They combine dead reckoning algorithms and RSS-based localization to define the positions of the individuals. They show that the introduction of RSS based localization improves their dead reckoning positioning errors for 50 to 60% after traveling 10 to 80 m. Their approach heavily depends of the traveled distance of the nodes; they have done several evaluations with up to 15 different nodes. For example, more than 40 m are needed in the combined approach to achieve errors that are smaller than 10 m, their best-case evaluation took 80 m for the nodes to travel but achieves positioning errors of 2.21 m, which is worse than our proposed MFAM.

Lately there has been a lot of research into light based indoor localization systems. These systems provide great accuracy, although they are not viable candidate for wide deployments, for example, Ma et al. [27] developed system with average error of 1.7 mm when localizing 56 points in 1 m^2^. Presented system cannot be efficiently scaled, as it requires a projector projecting images above the evaluation space. Similarly, Pandey et al. [28] evaluated nine points in the space of 9 m^2^, with six devices (light sources and reflection points) and obtained 0.3 m average error.

Authors of [29] promise centimeter accuracy indoor localization, while utilizing WiFi, yet they do not provide experiment or evaluation confirming the claim. They show how such accuracy can be achieved when defining position of transmitting AP in area of 0.14 m^2^ by a single receiving AP that is located in the neighboring room, while fingerprinting and sampling data are captured at the same time. Nevertheless, they show how the use of multiple frequency can be beneficial in estimation of the distances.

We have demonstrated that the fusion of multiple signals has multiple advantages; most importantly, it reduces the number of needed APs for each system for achieving low standard deviation of localization errors. Beneficial side effect is less interference and collisions while using these systems. This requires smaller investment in the deployment, particularly if other signals are already present in the buildings, as was the case in our evaluation environment. This further improves the real world applicability of our method. Furthermore, having different signals reduces the possibility of single-point failures thus improves the reliability of indoor localization, which can be very important for industrial usage.

Nowadays, if we want to implement even the simplest of the Wi-Fi-based localization method we need at a minimum three Wi-Fi APs. These usually implement 2.4 GHz Wi-Fi as well as high-speed 5 GHz. We usually cannot use the latter frequency, because we cannot provide coverage of all three 5 GHz signals throughout the indoor space; also, the signal is capable of wall penetration therefore, we cannot safely assume the room when detecting the signal. Future Wi-Fi APs will support 802.11ah standard, and therefore, additional 900 MHz Wi-Fi signal will be available throughout the indoor space. Therefore, it will be sensible to utilize those signals for indoor localization as well. With the three APs implementing both frequencies and multiple frequency approach to indoor localization, we can improve the localization accuracy over the usage of only 2.4 GHz. As expected, due to the difference in the network topology, we got worse results utilizing only 868 MHz signal. Nevertheless, we already showed the benefits and 18% improvements of the localization accuracy, while utilizing the multiple frequency approach.

## 6. Conclusions

We have presented MFAM, which is a novel indoor localization method, which utilizes different wireless frequencies to improve the localization accuracy. It reduces the number of required access points to simplify the deployment and improve real world applicability. Our method is purely model-based. It is based on the physical properties of RF signal propagation. It does not need any fingerprinting and does not require the devices to emit additional signals, which is important in real-world deployments. MFAM features self-calibrating operability, meaning that it can detect and adapt to the changes in the environment that have influence on the signal propagation, which improves the accuracy and makes our method less sensitive to changes in the indoors settings. Furthermore, our method is architecturally aware and foresees the inclusion of the floorplan into the method. This enables the localization with the respect to the walls and other obstacles. Our method is widely applicable and can be implemented using simple and accessible hardware.

We evaluated our method using 2.4 GHz Wi-Fi and 868 MHz home automation system. Wi-Fi-only evaluation shows better accuracy than similar methods, with an averaged median error of 2.65 m across four datasets. The evaluation results when using only the home-automation-system signals resulted in 16% worse average median error, which is still comparable to related non Wi-Fi methods, such as the Bluetooth method by [9], ZigBee method in [12] and FM based ACME [13].

When using multi-frequency approach of the MFAM, our results show considerably better accuracy than the other state-of-the-art methods with the averaged median accuracy of 2.14 m. The evaluation of our method shows the highest accuracy in places, which are not in close proximity of the wireless devices, which is important, as those places are often highly desirable for the accurate localization in real-world deployments.

We have achieved our goal of designing a localization method that can utilize future 802.11ah Wi-Fi standard designed for the specifics of the IoT. Although, due to the network topology of a gateway-based network, we were able to get only a quarter of the RSSI data when compared to the Wi-Fi, we showed 18% improvement of the localization accuracy is achievable in the real-world evaluation utilizing the multiple frequency approach of the MFAM.

In our future research, we are primarily interested into validation of the result on the 802.11ah hardware, when it will be available on the market. We are expecting better results, as the topology of the 802.11ah enables us to get more measurements than the gateway-based topology of the home automation system. More challenging research direction includes swapping frequency-specific devices of the Wi-Fi and home automation system with the software defined radio (SDR) modules. The SDR modules will enable us to capture wide frequency range of signals, therefore evaluating the proposed MFAM localization method on more signals simultaneously.

## Figures and Tables

**Table 1:** Comparison of the mean and median errors made by Multiple Frequency Adaptive Model-based localization (MFAM) method utilizing WiFi (2.4 GHz) signals.

Dataset	Mean Error [m]	Median Error [m]	SD [m]
DS1	2.50	2.55	1.31
DS2	2.43	2.55	1.21
DS3	2.89	2.55	1.62
DS4	2.77	2.69	1.91
Average	2.65	2.59	1.51

**Table 2:** Comparison of the mean and median errors made by MFAM utilizing HomeMatic (868 MHz) signals.

Dataset	Mean Error [m]	Median Error [m]	SD [m]
DS1	2.89	2.50	2.00
DS2	3.39	3.04	1.91
DS3	3.39	3.16	1.47
DS4	3.16	3.35	2.14
Average	3.21	3.01	1.88

**Table 3:** Comparison of the mean and median errors made by MFAM method combining WiFi (2.4 GHz) and HomeMatic (868 MHz) signals.

Dataset	Mean Error [m]	Median Error [m]	SD [m]
DS1	2.04	2.11	1.25
DS2	2.27	2.18	1.21
DS3	2.02	2.16	1.20
DS4	2.32	2.09	1.55
Average	2.16	2.14	1.30

## References

[1] NXP Laboratories UK (2016). ZigBee Light Link User Guide.

[2] International Telecommunication Union (ITU) (2012). Radio Regulations.

[3] Electronic Communications Committee (2015). ERC Recommendation 70-03.

[4] Tuta J., Juric M.B. (2016). A self-adaptive model-based Wi-Fi indoor localization method. Sensors.

[5] Yassin A., Nasser Y., Awad M., Al-Dubai A., Liu R., Yuen C., Raulefs R., Aboutanios E. (2017). Recent Advances in Indoor Localization: A Survey on Theoretical Approaches and Applications. IEEE Commun. Surv. Tutor..

[6] Bahl P., Padmanabhan V.N. RADAR: An in-building RF based user location and tracking system. Proceedings of the Nineteenth Annual Joint Conference of the IEEE Computer and Communications Societies (INFOCOM 2000).

[7] Kim Y., Chon Y., Cha H. (2012). Smartphone-based collaborative and autonomous radio fingerprinting. IEEE Trans. Syst. Man Cybern. Part C Appl. Rev..

[8] Lim H., Kung L.C., Hou J.C., Luo H. Zero-configuration, robust indoor localization: Theory and experimentation. Proceedings of the IEEE INFOCOM.

[9] Tarrío P., Bernardos A.M., Casar J.R. (2011). Weighted Least Squares Techniques for Improved Received Signal Strength Based Localization. Sensors.

[10] Du Y., Yang D., Xiu C. (2015). A novel method for constructing a WIFI positioning system with efficient manpower. Sensors.

[11] Jin M.-H., Yu C.-H., Lai H.-R., Feng M.-W., Thulasiraman P., He X., Xu T.L., Denko M.K., Thulasiram R.K., Yang L.T. (2007). Zigbee Positioning System for Smart Home Application. Frontiers of High Performance Computing and Networking ISPA 2007 Workshops, Proceedings of the ISPA 2007 International Workshops SSDSN, UPWN, WISH, SGC, ParDMCom, HiPCoMB, and IST-AWSN, Niagara Falls, ON, Canada, 28 August–1 September 2007.

[12] Shimosaka M., Saisho O., Sunakawa T., Koyasu H., Maeda K., Kawajiri R. (2016). ZigBee based wireless indoor localization with sensor placement optimization towards practical home sensing*. Adv. Robot..

[13] Yoon S., Lee K., Yun Y.C., Rhee I. (2016). ACMI: FM-based indoor localization via autonomous fingerprinting. IEEE Trans. Mob. Comput..

[14] Zhuang Y., Yang J., Li Y., Qi L., El-Sheimy N. (2016). Smartphone-Based Indoor Localization with Bluetooth Low Energy Beacons. Sensors.

[15] Hossain A.K.M.M., Van H.N., Jin Y., Soh W.-S. Indoor Localization Using Multiple Wireless Technologies. Proceedings of the IEEE International Conference on 2007 Mobile Adhoc and Sensor Systems.

[16] Laufer S., Mallas C. Attacking HomeMatic. https://www.youtube.com/watch?v=CWcpJ73j-GQ.

[17] Lui G., Gallagher T., Li B., Dempster A.G., Rizos C. Differences in RSSI readings made by different Wi-Fi chipsets: A limitation of WLAN localization. Proceedings of the 2011 International Conference on Localization and GNSS (ICL-GNSS).

[18] (2016). Standards Committee of the IEEE Computer Society 802.11-2016—IEEE Standard for Information Technology—Telecommunications and Information Exchange between Systems Local and Metropolitan Area Networks—SPECIFIC Requirements—Part 11: Wireless LAN Medium Access Control (MAC) and Physical Layer (PHY).

[19] European Commision COST Action 231 (1999). Digital Mobile Radio towards Future Generation Systems—Final Report.

[20] Xu L., Yang F., Jiang Y., Zhang L., Feng C., Bao N. Variation of Received Signal Strength in Wireless Sensor Network. Proceedings of the 2011 3rd International Conference on Advanced Computer Control.

[21] Bose A., Chuan H.F. A practical path loss model for indoor WiFi positioning enhancement. Proceedings of the 2007 6th International Conference Information, Communication Signal Processing (ICICS 2007).

[22] Kanaris L., Kokkinis A., Liotta A., Stavrou S. (2017). Fusing Bluetooth Beacon Data with Wi-Fi Radiomaps for Improved Indoor Localization. Sensors.

[23] Ruan W., Sheng Q.Z., Yao L., Li X., Falkner N.J.G., Yang L. (2018). Device-free human localization and tracking with UHF passive RFID tags: A data-driven approach. J. Netw. Comput. Appl..

[24] Li X., Yang Y., Cai J., Deng Y., Yang J., Zhou X., Tan L. (2018). Plils: A Practical Indoor Localization System through Less Expensive Wireless Chips via Subregion Clustering. Sensors.

[25] Seco F., Jimenez A.R. (2018). Smartphone-Based Cooperative Indoor Localization with RFID Technology. Sensors.

[26] Noh Y., Yamaguchi H., Lee U. (2018). Infrastructure-Free Collaborative Indoor Positioning Scheme for Time-Critical Team Operations. IEEE Trans. Syst. Man Cybern. Syst..

[27] Ma S., Liu Q., Sheu P.C.-Y. (2018). Foglight: Visible Light-Enabled Indoor Localization System for Low-Power IoT Devices. IEEE Internet Things J..

[28] Pandey O.J., Sharan R., Hegde R.M. (2017). Localization in Wireless Sensor Networks Using Visible Light in Non-Line of Sight Conditions. Wirel. Pers. Commun..

[29] Chen C., Chen Y., Han Y., Lai H.-Q., Liu K.J.R. (2016). Achieving Centimeter Accuracy Indoor Localization on WiFi Platforms: A Frequency Hopping Approach. IEEE Internet Things J..

